# Unraveling the Complexity of Plant Trichomes: Models, Mechanisms, and Bioengineering Strategies

**DOI:** 10.3390/ijms26147008

**Published:** 2025-07-21

**Authors:** Tiantian Chen, Yanfei Ma, Jiyan Qi

**Affiliations:** 1 Shaanxi Key Laboratory of Qinling Ecological Intelligent Monitoring and Protection, School of Ecology and Environment, Northwestern Polytechnical University, Xi’an 710129, China; ttchen@nwpu.edu.cn; 2 Shenzhen Research Institute, Northwestern Polytechnical University, Shenzhen 518057, China; 3 Collaborative Innovation Center, Northwestern Polytechnical University, Shanghai 201108, China

**Keywords:** glandular secretory trichome, plant natural products, metabolism

## Abstract

Trichomes—microscopic appendages on the plant epidermis—play vital roles as both protective barriers and specialized biosynthetic factories. Acting as the first line of defense against environmental stressors, they also produce a wide range of pharmaceutically valuable secondary metabolites. This mini-review highlights recent advances in understanding the development, structure, and function of trichomes, with a focus on glandular secretory trichomes (GSTs) in key species such as *Artemisia annua* and *Solanum lycopersicum*. We explore how insights from these systems are driving innovation in plant synthetic biology, including modular genetic engineering and metabolic channeling strategies. These breakthroughs are paving the way for scalable, plant-based platforms to produce high-value compounds. By integrating molecular mechanisms with emerging technologies, this review outlines a forward-looking framework for leveraging trichomes in sustainable agriculture, natural product discovery, and next-generation biomanufacturing.

## 1. Introduction

Trichomes are specialized outgrowths of the plant epidermis that exhibit remarkable morphological diversity—including unicellular, multicellular, stellate, glandular, and hooked forms. They are widely distributed across plant organs such as stems, leaves, flowers, fruits, and seeds in species like *Artemisia annua* and *Solanum lycopersicum* [1]. These structures play critical roles in plant defense, water retention, and temperature regulation—functions honed through long evolutionary adaptation. Beyond their ecological significance, trichomes offer a powerful model for studying fundamental biological processes such as cell differentiation, signal transduction, and environmental responsiveness [2]. Importantly, many trichomes, especially glandular types, serve as biochemical factories that produce secondary metabolites essential for agriculture, medicine, and industry. These compounds contribute to pest resistance, human health, material innovation, and environmental resilience [1,2]. An in-depth examination of plant trichomes is crucial to unlocking the potential of plants, as well as serving the sustainable development of society.

## 2. Epidermal Appendage

**Figure 1:**
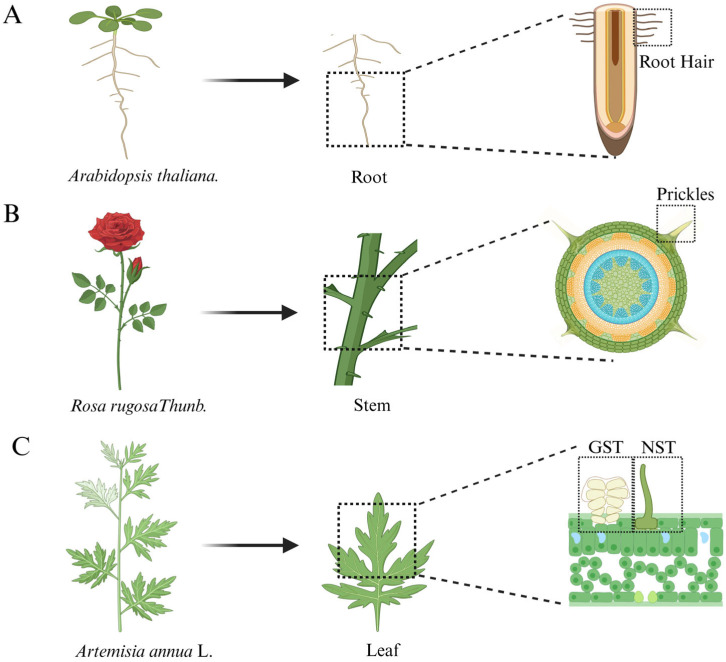
Epidermal appendages in plants. (**A**) Root epidermal cells differentiate into root hairs [3]. (**B**) Stem epidermal cells differentiate into prickles [4]. (**C**) Leaf epidermal cells differentiate into trichomes [5] (glandular secretory trichome (GST) and non-glandular trichome (NGT)). Image created with BioRender.com, URL (accessed on 11 July 2025) used with permission.

The plant epidermis is a protective layer covering the surface of seeds, roots, stems, leaves, flowers, and fruits. In addition to trichomes, epidermal cells also produce special structures, such as root hairs and prickles, that serve a variety of functions (Figure 1), including defenses and secretions of bioactive compounds [1,2].

### 2.1. Root Hairs: Plant Growth and Intercellular Signaling

Root hairs, which are specialized appendages derived from root epidermal cells, play a crucial role in the regulation of plant growth and intercellular communication. Cells called root hair cells are responsible for the development of these structures, while adjacent cells lacking this capacity are referred to as non-hair cells [3]. Based on the spatial patterning of root hairs and non-hair cells, plants can be classified into two broad categories: 1. A monocotyledonous plant (such as rice, *Oryza sativa*) is able to differentiate all epidermal cells into root hairs without regard to their location [6]. 2. Species with position-dependent root hair patterning; for example, *Brachypodium distachyon* has two distinct cell types, large and small. Small cells differentiate into root hairs [7]. Dicotyledonous plants, such as *Arabidopsis thaliana*, alternate root hair cells with non-hair cells along the epidermis [3]. Root hair development in Arabidopsis is regulated by the core regulatory complex WER-GL3/EGL3-TTG1, which promotes the expression of GL2 and ZP1. A variety of plant hormones are also involved in root hair formation, including ethylene, cytokinin, auxin, BR, and JA [8].

### 2.2. Prickles: Morphological Adaptations and Functional Diversity

Prickles evolved as sharp epidermal structures and primarily function in deterring herbivores [9]. Research indicates that the prickles of rubus and roses are modified trichomes that grow and harden eventually, growing outward from the epidermis [4,10]. A new classification has been proposed for prickles within the *Rosaceae* family, based on their secretory capability. There are two types of prickles, non-glandular prickles (NGPs) and glandular prickles (GPs) [5]. A complex process is involved in the development of prickles. Through QTL analysis, recent research has identified a major locus on LG3 that influences the development of prickles on rose stems. Further investigation and refinement of the regulatory network are still in progress [11].

### 2.3. Trichomes: Non-Glandular and Glandular Variants

Trichomes are the most prominent epidermal appendages. They display a remarkable diversity of function and structure [12]. In terms of their ability to synthesize and secrete metabolites, they can be broadly categorized into non-glandular trichomes (NGTs) and glandular secretory trichomes (GSTs) [13] (Table 1).

NGTs are found in angiosperms, gymnosperms, and mosses and function primarily to deter herbivores and insects. Cotton fibers are an example of a specialized non-glandular trichome found in cotton plants that provides pest resistance against *Silverleaf whiteflies* [14]. As a result of their ability to form physical barriers, NGTs play a crucial role in protecting plants from external threats.

The GSTs, on the other hand, are specialized in synthesizing, secreting, or storing a wide variety of secondary metabolites with significant ecological, economic, and medicinal applications, as well as serving as a defense against external pathogens and pests [15]. *Cannabis sativa*, *Solanum lycopersicum*, and *Artemisia annua* exhibit a wide variety of trichome morphologies, ranging from stalked and bulbous structures to multicellular structures. Among the most effective malaria drugs, artemisinin is synthesized in the glandular secretory trichomes of *Artemisia annua* [16]. Similarly, cannabis glandular secretory trichomes produce cannabinoids, including tetrahydrocannabinol (THC) and cannabidiol (CBD), which have a number of therapeutic uses [17].

In addition, GSTs are capable of producing menthol from mint, phenolic compounds from rosemary, and volatile oils from *Schizonepeta tenuifolia* [18]. The tomato GSTs (type IV) secrete sticky substances that trap fungal gnats and immobilize small arthropods, demonstrating their dual roles in plant defense and ecology [19].

## 3. Non-Glandular Trichomes: A Model System for Studying Cell Differentiation

### 3.1. Structure and Morphology Characteristics of NGTs

Non-glandular trichomes have a relatively simple structure, with most being unicellular in nature. Arabidopsis has typical unicellular, non-glandular trichomes with two or three branches [20]. The development of non-glandular trichomes in Arabidopsis has been extensively studied, making it a key system for understanding epidermal cell fate determination. A trichome′s development typically involves initiation followed by four rounds of endoreplication and branching. Natural resources such as cotton fiber, which is composed of unique unicellular trichomes that arise from the seed coat epidermis, are vital for the textile industry [21]. There are four distinct stages in their development: (1) initiation, (2) elongation (primary cell wall synthesis), (3) secondary cell wall synthesis, and (4) maturation.

### 3.2. Biological Functions of NGTs

The protruding structure of NGTs contributes to the isolation of the epidermis from the external environment, thus protecting the plant from some of the effects of environmental factors. Recently, research has demonstrated that trichomes are important for the practical production of many economic crops. In soybeans (*Glycine max*), epidermal trichomes, commonly referred to as pubescence, are unbranched, single-celled structures. It has been demonstrated that trichome density correlates strongly with traits such as drought resistance and insect resistance in soybeans, demonstrating their importance both ecologically and agriculturally. Among the *Brassicaceae* family, mustard (*Brassica juncea*) is an important crop that serves as an ingredient in condiments, leafy vegetables, and edible oils. Palatability is influenced by the density and morphology of its unicellular, non-glandular, unbranched trichomes. Furthermore, these epidermal structures serve as a primary defense mechanism against insect herbivory [22].

### 3.3. Regulatory Mechanism of NGTs Development

Arabidopsis trichome development is regulated by the MYB-bHLH-WD40 (MBW) protein complex. Through this regulatory network, trichome differentiation and development are orchestrated, providing insight into the genetic pathways underlying cell fate determination [20]. Due to the economic importance of cotton fiber, many transcription factors have been implicated in its development, including the MYB, bHLH, and HD-ZIP transcription factors [21]. MYB25 and HOX3 have recently been identified as critical regulators of fiber differentiation, as well as the single-cell tip-biased diffusion of growth [23]. In soybeans, three genes are responsible for regulating pubescence density. *Pd1* encodes a transcription factor of the HD-ZIP IV class. *Ps* encodes a multidomain protein that contains evolutionarily conserved WD40 and RING domains, which are central to its structural configuration and mediate specific interactions between proteins. *Mao1* plays an important role in controlling the morphology of soybean epidermal trichomes. As central regulators of pubescence density, these genes work in a feedback loop to maintain optimal trichome density, balancing defense and physiological efficiency [24]. The morphology of soybean trichomes has evolved significantly from its wild ancestors. It is noted that wild soybeans possess inverted, flat trichomes, whereas cultivated varieties possess upright, slightly angular trichomes. Ty3/Gypsy retrotransposon insertions in the *Mao1* promoter result in increased *Mao1* expression, leading to upright trichome development. By overexpressing *Mao1* in soybean, upright trichomes can be developed, improving light capture and photosynthetic efficiency, which results in higher yields [25]. In *Brassica juncea*, *BjGL1b1* (an *AtGL1* homolog) serves as a key regulator of the development of non-glandular trichomes [26]. In contrast, the HD-Zip III transcription factor *BjPHVa* negatively modulates the development of trichomes [27].

Arabidopsis and cotton are well-established models for studying non-glandular trichomes. Over the past five years, researchers have observed a significant increase in yield potential and commercial value in the trichomes of economically important crops, such as soybeans (*Glycine max*) and mustards (*Brassica juncea*). It has been demonstrated that HD-Zip transcription factors are evolutionarily conserved to regulate NGT development across species, which offers significant implications for future research on trichome biology in other cash crops.

## 4. Glandular Secretory Trichomes (GSTs): Natural Biofactories

In comparison with NGTs, GSTs exhibit greater complexity in terms of structural morphology, synthetic function, and transcriptional regulation. Thus, studies on single-cell non-glandular trichomes in Arabidopsis and cotton served as a reference at the onset of GSTs research.

### 4.1. Structure and Morphology Characteristics of GSTs

**Figure 2:**
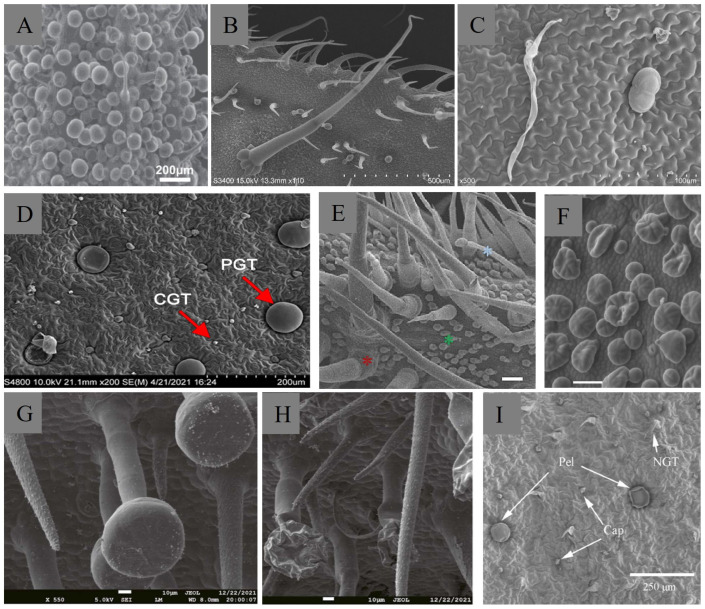
The morphology of glandular secretory trichomes in different species. GSTs of (**A**) *Cannabis sativa* [37], (**B**) *Solanum lycopersicum*, (**C**) *Artemisia annua*, (**D**) *Thymus vulgaris* [35], (**E**) *Cucumis sativus* [33], different types of trichomes marked by red, blue, green asterisks, (**F**) *Phillyrea latifolia* [32], (**G**,**H**) *Lonicera japonica* [36], and (**I**) *Schizonepeta tenuifolia* [18].

GSTs develop in a pattern that is derived from protodermal cells. These cells protrude outward and divide periclinally to form stalk cells and apical head cells. Head cells divide differently depending on the shape of the stalk cells, resulting in varying types of glandular secretory trichomes [15]. The heads of glandular secretory trichomes are the secretory parts, usually spherical, ovoid, cylindrical, or other shapes, where secretions are produced and stored. The stalks support and nourish the head by connecting it to the epidermal cells, which may be short and thick or long and thin [5,15,19]. The diversity of GSTs is a result of dual selection pressures from the environment and pests, which causes them to secrete specific secondary metabolites (Figure 2).

*Cannabis* is an herbaceous plant that has been consumed and used medicinally for centuries. In *Cannabis*, GSTs play a central role in plant defense by producing and storing secondary metabolites such as cannabinoids. These compounds not only deter insect pests and suppress pathogen invasion but also enhance the plant’s resilience to abiotic stresses, including drought and ultraviolet radiation [13]. *Cannabis* contains three types of GSTs: capitate-stalked GTs, capitate-sessile GTs, and bulbous GTs. A capitate-stalked GT contains 12–16 disc cells, while a capitate-sessile GT contains 8 disc cells. Bulbous GT, the smallest type, consists of a bulbous head with a minimal stalk structure [28]. *Cannabis* is a dioecious species with marked sexual dimorphism in GST distribution, with female plants exhibiting significantly higher GST density levels, which exhibit a preferential accumulation in reproductive structures, particularly bracteal structures and floral calyxes [29]. *Artemisia annua* is able to adapt to different environmental conditions due to its secretory function. In drought or high-temperature conditions, volatile oils and waxy substances secreted by the GSTs may reduce water loss and enhance the plant′s stress tolerance [16]. *Artemisia annua* GSTs consist of ten cells arranged in a double row: two basal cells, two stem cells, four subapical cells, and two apical cells. A wax vesicle is surrounded by two pairs of apical secretory cells, and the space between them is known as the subepidermal space [30,31]. In tomatoes, research has found that the various types of trichomes can repel insect pests, trap them physically, and attract natural enemies for indirect defense [19]. Tomato trichomes are classified into eight types, of which types I, IV, VI, and VII are glandular, containing terminal glands that secrete and store various secondary metabolites, particularly terpenoids [19]. In the GSTs of *Ocimum basilicum*, the phenylpropanoid pathway has been shown to be capable of synthesizing eugenol, chavicol, and their methylated derivatives. *Phillyrea latifolia* also accumulates flavonoid glycosides within its glandular secretory trichomes as a means of protecting itself against ultraviolet radiation [32]. *Cucurbitaceae* cucumber plants possess two different types of glandular secretory trichomes: types I and VI. Type I consists of three to four stalk base cells and four to five glandular cells within the head. The stalk base of type VI is longer than that of type I. The variability of stalk length and thickness allows GSTs to adapt to various environments in different ways [33,34]. Thyme is a perennial herb or half-shrub in the *Lamiaceae* family. The high levels of active ingredients in thyme give it certain medicinal properties, including antioxidant, antibacterial, and anticancer properties. Two types of GSTs are found on the leaf blades of thyme: peltate glandular trichomes (PGTs), capitate glandular trichomes (CGTs), and non-glandular trichomes (NGTs). Terpenoids produced in PGTs are responsible for their medicinal properties [35]. Honeysuckle refers to the dried flower buds or partially opened flowers of *Lonicera japonica* Thunb., a plant belonging to the Caprifoliaceae family and the *Lonicera genus*. The flowers and stems of honeysuckle are both used medicinally in traditional Chinese medicine. It is effective against colds, headaches, fevers, rashes, influenza viruses, and other ailments. *Lonicera japonica* GSTs are all capitated in structure. They consist of a stalk cell, a basal cell that is attached to the stalk, and a multicellular disc that surrounds the basal cell. *LjROC3*, an HD-ZIP gene, increases the density of GSTs on honeysuckle leaves [36].

### 4.2. Secondary Metabolite Synthesis in GSTs

As biosynthetic hubs, GSTs produce and secrete a variety of volatile and non-volatile substances, such as terpenes, phenolics, lipids, sugars, and alkaloids. Artemisinin and cannabinoids are well-known secondary metabolites derived from GSTs for their significant medicinal effects [12,17]. Cannabinoids are terpenophenolic compounds that constitute the primary active constituents of *Cannabis*. These compounds have been shown to possess anti-epileptic, anti-depressant, neuroprotective, anti-inflammatory, analgesic, and anti-tumor properties [28,29]. There are four key stages in the biosynthesis of cannabinoids: hexanoate biosynthesis, 2-methylerythritol-4-phosphate (MEP) biosynthesis, geranylpyrophosphate (GPP) biosynthesis, and cannabinoid biosynthesis. The cannabinoid pathway utilizes products synthesized from the preceding three pathways as substrates. Specifically, olivetol synthase (OLS) catalyzes the condensation of hexanoyl-CoA (derived from the hexanoate pathway) with malonyl-CoA to form olivetolic acid (OA). In the next step, olivetolic acid undergoes prenylation, resulting in cannabigerolic acid (CBGA) by combining with GPP [38]. It is believed that this step is the most crucial in the process of producing cannabinoids. Additionally, CBGA serves as the central precursor and is specifically catalyzed by enzymes found in GSTs: tetrahydrocannabinolic acid synthase (THCAS), cannabidiolic acid synthase (CBDAS), and cannabichromenic acid synthase (CBCAS). This catalysis produces tetrahydrocannabinolic acid (THCA), cannabidiolic acid (CBDA), and cannabichromenic acid (CBCA), respectively. Finally, these acidic cannabinoids undergo non-enzymatic decarboxylation (typically through heating) to yield the neutral forms: Δ^9^-tetrahydrocannabinol (THC), cannabidiol (CBD), and cannabichromene (CBC).

Malaria, known as one of the most life-threatening diseases on earth, is an infectious disease caused by Plasmodium parasites. It is transmitted to humans through the bites of infected female Anopheles mosquitoes. Artemisinin, a sesquiterpene lactone compound, was first discovered and extracted from the plant *Artemisia annua* in 1972 by Tu Youyou, based on traditional Chinese medicine knowledge [39]. Artemisinin-based combination therapies (ACTs) have been recommended by the World Health Organization (WHO) as the most effective treatment for malaria at present [40]. Artemisinin is synthesized in subapical and apical cells, and it accumulates in the subepidermal space surrounding wax vesicles. Artemisinin′s biosynthetic pathway has been extensively investigated over the past decade. Artemisinin is synthesized via the isoprenoid biosynthetic pathway, which utilizes farnesyl pyrophosphate (FPP) and its precursor, isopentenyl diphosphate (IPP). In plants, IPP is produced through two distinct pathways: the cytoplasmic mevalonate (MVA) pathway and the plastid-localized methylerythritol phosphate (MEP) pathway. Amorpha-4,11-diene synthase (ADS) catalyzes the cyclization of FPP to form amorpha-4,11-diene [41], which is recognized as the first committed step in artemisinin biosynthesis. Amorpha-4,11-diene undergoes three sequential hydroxylations catalyzed by the cytochrome P450 multifunctional oxidase CYP71AV1, yielding artemisinic alcohol, artemisinic aldehyde, and (finally) artemisinic acid [42]. Artemisinic aldehyde is then reduced by artemisinic aldehyde Δ11(13) reductase (DBR2) to form dihydroartemisinic aldehyde. This intermediate is oxidized by aldehyde dehydrogenase 1 (ALDH1) to produce dihydroartemisinic acid, the primary precursor of artemisinin. However, the mechanism by which dihydroartemisinic acid is converted into artemisinin in the final reaction step remains unclear.

### 4.3. Regulatory Mechanism of GSTs Development

Although there are many plants containing GSTs, given the identification of complete genomes and plant genetic transformation systems, most research has focused on *Artemisia annua* and multicellular tomato GSTs; in recent years, GST transcriptional regulatory networks have been further refined, and significant advances have been made in cellular information exchange and cell fate determination.

*Cannabis*′ regulatory network for GT development has not yet been explored. In recent studies, transcriptomics was analyzed in 8-week-old female flowers of *Cannabis sativa* and revealed that GT development is a highly coordinated process regulated at the transcriptional level by the WRKY, MYB, bHLH, and ERF families. *CsMYC4* was identified as a key gene that controls GT formation through MeJA signaling [37]. The molecular regulation of the glandular secretory trichomes of *Artemisia annua* has been elucidated in recent studies. It has been determined that the HD-ZIP IV transcription factors AaHD1 and AaHD8, which have been identified in *Artemisia annua*, positively regulate GST production. In addition, AaHD8 promotes the expression of *AaHD1* via jasmonic acid (JA) signaling [31,43]. GST initiation is activated by the JA-responsive transcription factor AaSPL9 [44]. R2R3-MYB AaMYB17, which is expressed in shoot tip trichomes, is a GST-specific positive regulator [45]. In GST basal cells, AaMIXTA1 plays a critical role in promoting both GST initiation and artemisinin synthesis, while regulating the synthesis of leaf cuticles [46]. AaGSW2, a GST-specific factor, is directly activated by AaHD1/AaHD8; its overexpression increases GST density and artemisinin concentrations [47]. Through interaction with AaMIXTA1 and the indirect upregulation of AaGSW2, AaWIN1 increases GST density [48]. In contrast, AaMYB16 and AaMYB5 are negative regulators. AaGSW2 is not directly controlled by either of these genes, but both affect AaHD1′s binding affinity to AaGSW2 [30]. MADS-box factor AaSEP1 promotes GST initiation by integrating JA and developmental signals. By interacting with AaMYB16, it amplifies the AaHD1-mediated activation of the AaGSW2 promoter, thereby increasing GST density and the production of artemisinin [49]. AaTAR2, a R2R3-MYB factor, also promotes GST development and terpene/flavonoid synthesis. Its expression is directly enhanced by AaHD1 and AaHD8 [50]. AaMYB1 is a member of the S13 subfamily and induces GST formation by activating GL1 and GL2, which also affect gibberellin GA4 metabolism [51]. Conversely, the AaTLR1–AaWOX1–AaTLR2 complex suppresses GST development through GA3 regulation [52]. In addition, TRICHOME AND ARTEMISININ REGULATOR 1 (TAR1) binds to the promoters of CYP71AV1 and ADS, coregulating the development of GST and artemisinin production [53].

Tomato type I, IV, VI, and VII trichomes are GSTs. Wo, the key gene for tomato GST formation, was initially identified by map-based cloning. Woolly mutants (LA3186) have dense trichome layers due to gain-of-function mutations [54]. Wo gene protein–protein interactions facilitate the transition of cells from the G2 to M phase, thereby promoting epidermal trichome formation [55]. Further studies have been conducted over the past decade to examine the roles of related transcription factors in regulating the differentiation of different types of trichomes in tomatoes. These transcription factors include those associated with the R2R3-MYB, HD-Zip IV, C2H2, and bHLH gene families [56,57,58]. Studies demonstrate that applying exogenous jasmonic acid (JA) to developing tomato leaves increases type VI glandular trichome density, enhancing secretory capacity and reducing thrips damage [59]. The JA signaling repressor SlJAZ2 inhibits glandular trichome initiation by suppressing the expression of SlWo and SlCycB2 [60]. GST development (types I + VI) in tomato is positively regulated by the F-box protein SlCOI. Given the direct SlJAZ2-SlCOI interaction, the JA-induced proteasomal degradation of SlJAZ2 likely facilitates trichome formation [61]. JA treatment significantly elevates trichome density (types I + II) and terpenoid synthesis. Under JA conditions, zinc finger proteins H and H-like (HL) coregulate glandular trichome formation (type I). The SlJAZ2 protein physically interacts with H/HL to suppress their activity, thereby activating THM1, a trichome repressor [58]. Loss-of-function in the HD-ZIP IV transcription factor Woolly (Wo) impairs multiple epidermal trichome types (including type VI) and terpene content. Wo activates terpenoid biosynthesis by binding TPS gene promoters and recruiting the terpene regulator SlMYC1. SlJAZ2 competitively binds both Wo and SlMYC1, disrupting Wo/SlMYC1 complex formation and inhibiting TPS transcription [57]. Methyl jasmonate (MeJA) treatment enlarges type IV trichome glandular cells. The trichome-enriched HD-ZIP IV factor SlHD8 interacts with SlJAZ4; SlHD8 knockdown causes trichome shortening. SlHD8 directly activates five cell-wall-loosening enzymes to regulate elongation. Thus, JA promotes trichome elongation via the SlJAZ4-SlHD8 module, which modulates cell wall remodeling [62]. SlbHLH95 acts as a negative trichome regulator (type I) by directly binding gibberellin biosynthesis gene promoters (SlGA20ox2, SlKS5) [56]. Auxin signaling also contributes to tomato trichome initiation. The auxin response factor SlARF3, crucial for auxin transduction, is essential for epidermal development: SlARF3 knockdown reduces pavement cell density and type I/V/VI trichomes [63]. Similarly, SlIAA15 (another auxin response factor) participates in trichome formation; its downregulation decreases type I and VI trichome density [64]. Despite these findings, it remains unclear how genes regulate the initiation of epidermal trichomes and how they differentiate into different types of epidermal trichomes. Recent research has suggested that the Wo protein regulates epidermal cell differentiation into digitate trichomes (DTs) or peltate trichomes (PTs) by precisely controlling the dosage [65]. A marked disparity in Wo protein dosage has been observed between DTs and PTs, with DTs exhibiting substantially elevated levels compared to PTs. How does the Wo protein concentration regulate glandular secretory trichome differentiation? Two candidate regulators, SlWox3b (WOX family) and MX1 (MYB domain), have shown expression patterns strongly correlated with Wo protein abundance across tomato genotypes. In one study, in SlWox3b and MX1 mutants, DTs decreased and PTs increased, but total glandular secretory trichomes were not different from controls. ChIP-seq analysis and RNA-seq analysis of Wo, SlWox3b, and MX1 genetic plants show that the LFS gene regulates PTs formation. Moreover, knocking out the SlWox3b, MX1, and LFS genes simultaneously results in tomato epidermal trichomes remaining at the single-cell stage and unable to differentiate further. PTs or DTs are differentiated by Wo protein concentration changes. This study elaborates on the view that tomato epidermal trichome cells originate from the same cell lineage from the perspective of cell development for the first time. According to the latest research, epidermal trichome development is also closely linked to tomato reproduction. Several HD-Zip IV transcription factors are highly expressed in cells that initiate the clasp-like epidermal trichomes on tomato anthers, and they regulate the initiation and endoreduplication of these trichomes. These clasp-like epidermal trichomes contribute to tomatoes′ closed anther tube development through long-term cultivation and domestication [66].

**Figure 3:**
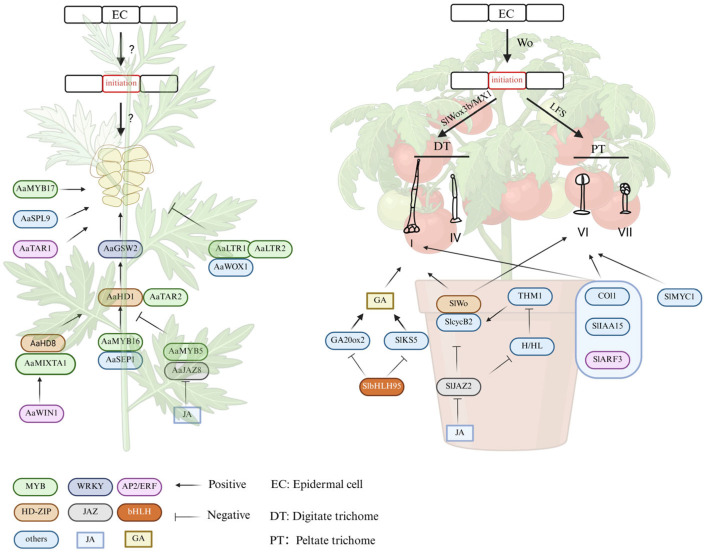
Model of glandular secretory trichome development in *Artemisia annua* and *Solanum lycopersicum*. Image created with BioRender.com, URL (accessed on 11 July 2025) used with permission.

In recent years, advancing research has led to progressively deeper insights into the development and molecular regulation of glandular secretory trichomes (GSTs) in model species such as *Artemisia annua* and tomatoes (Figure 3). Notably, recent studies on the WO protein in tomatoes have shed light on trichome development at the cellular level, offering a detailed view of the underlying genetic control. In contrast, investigations into GST development and regulatory genes in other important species, such as thyme and honeysuckle, are still in their early stages.

To fully realize the potential of GSTs, future research must further explore the transcriptional networks and cell fate determination pathways that govern their formation. A deeper understanding of these mechanisms will not only advance our knowledge of plant developmental biology but also provide critical guidance for the breeding and metabolic engineering of horticultural crops, medicinal plants, and other economically important species.

## 5. Applications and Future Perspectives

Glandular secretory trichomes (GSTs) are a striking example of evolutionary innovation, uniquely combining a specialized cellular architecture with a remarkable biosynthetic capacity. Across both model and non-model plant species, research has revealed conserved regulatory modules—such as the HD-ZIP IV and R2R3-MYB transcription factors—as well as diverse structural adaptations that support their dual role in plant defense and the production of bioactive secondary metabolites.

The application value of trichomes in agriculture and medicine is wide-ranging. There is considerable potential for the future medical and agricultural applications of specialized metabolites that are biosynthesized by GSTs. These products are known to be important reservoirs of high-value bioactive natural products. Due to the persistent limitations of genetic transformation techniques, current studies remain focused on *Artemisia annua and Solanum lycopersicum*. However, the genetic research underlying their development is still in its infancy. Research on trichomes faces significant challenges in two areas: (1) elucidating how epidermal cells differentiate into trichomes—a complex process governed by hormonal signals, transcriptional networks, and environmental cues, and (2) improving the metabolic efficiency of GSTs by identifying rate-limiting biosynthetic enzymes and transporters involved in metabolite accumulation and secretion.

Synthetic biology has emerged as a powerful tool in biotechnology, enabling the synthesis of drugs, nutrients, and other industrial products in heterologous systems [67]. A notable example is the production of artemisinic acid in yeast, which demonstrates the potential of microbial systems to synthesize valuable compounds. However, challenges such as high production costs and low yield currently hinder its commercialization [68]. While microbial platforms have dominated synthetic biology, plants offer unique advantages as chassis due to their ability to perform photosynthesis and their positive environmental impact. As a result, plant synthetic biology is gaining increasing attention as an emerging field [69,70]. Despite this potential, the use of plants as synthetic biology platforms remains limited. This is primarily due to plants′ relatively slow growth, the complexity of gene regulatory networks, and the challenges associated with genetic manipulation. Addressing these obstacles is essential to unlocking plants′ full potential in synthetic biology applications. Despite the success of plant systems like tomatoes, mosses, and tobacco in producing highly valuable metabolites and therapeutic proteins, significant knowledge and technical gaps remain [70,71]. The key challenges include identifying mechanisms underlying the efficient synthesis and storage of secondary metabolites, as well as unraveling the mechanisms underlying the development of specialized structures such as GSTs [72]. Further, it is essential to examine how trichome traits can be improved in the major crops in order to develop cultivars with a strong defense against herbivorous pests. With the aid of this approach, epidermal trichomes will become a tunable regenerative tool that will be used to tailor breeding programs for different species according to their specific agricultural requirements.

Emerging technologies such as single-cell transcriptomics and genome editing are poised to resolve spatial heterogeneity in metabolite biosynthesis, identify rate-limiting regulators, and ultimately allow for rational trichome reprogramming. These advances could transform underutilized medicinal plants into optimized platforms for sustainable bioactive compound production. The single-cell RNA-seq (scRNA-seq) method offers a powerful solution to these challenges, resolving biosynthesis pathways and understanding cellular development trajectories with individual-cell precision. In *Catharanthus roseus*, scRNA-seq has been instrumental in uncovering the multicellular compartmentalization of secondary metabolism, revealing the multicellular compartmentalization phenomenon in catharanthine biosynthesis [73,74]. Similarly, its application in Limonium bicolor revealed the intricate developmental regulation of salt glands, demonstrating the utility of single-cell technologies in decoding specialized plant structures [75]. These studies have identified novel targets and strategies for enhancing active ingredient production through molecular-assisted breeding and heterologous synthesis. By using single-cell technology, we expect to be able to elucidate the complete developmental trajectory of glandular trichomes and their dynamic biosynthetic progression, leading to major breakthroughs in synthetic biology and cultivar development. These advances will enhance production efficiency and drive industrial-scale applications, promoting the commercialization and accessibility of high-value metabolites.

To move from descriptive biology to predictive and translational design, future trichome research must integrate developmental genetics, spatial omics, synthetic pathway design, and ecological validation. GSTs offer not only a window into plant adaptive strategies but also a chassis for next-generation green factories—blurring the boundary between natural evolution and engineered biosynthesis.

## 6. Conclusions

In recent years, interest in trichomes has steadily increased, driven particularly by advances in research on multicellular GSTs. While previous studies have summarized factors influencing the development of plant trichomes and secondary metabolite synthesis, their reviews lacked comprehensive summaries. This review summarizes the latest advances in NGTs in crop production, further refines the research findings on GSTs in *A. annua* and tomatoes at the cellular development level, and proposes the application of single-cell technology in the study of multicellular GSTs. We expect that this mini-review will provide a valuable reference for future fundamental and applied research on plant trichomes.

## Figures and Tables

**Table 1:** Overview of trichome types, morphology, and biological functions in representative plant species.

Plant Species	Trichome Types	Trichome Morphology	Major Biological Function(s)
*Arabidopsis thaliana*	NGT	unicellular branched	Physical barrier defense
*Gossypium spp.*	NGT	unicellular unbranched	Cellulose storage (commercial fiber)
*Glycine max*	NGT	unicellular unbranched	Enhanced photosynthetic efficiency
*Brassica juncea*	NGT	unicellular unbranched	Defense-related responses
*Cannabis sativa*	GST	Multicellular: capitate, bulbous	Secretion of cannabinoids (THC/CBD)
*Solanum lycopersicum*	GST	Multicellular: digitate, peltate	Secretion of acyl sugars, insect repellence
*Artemisia annua*	GST	Multicellular: peltate	Artemisinin secretion (antimalarial)
*Schizonepeta tenuifolia*	GST	Multicellular: peltate, capitate	Secretion of menthol-rich essential oils (antibacterial, insecticidal)
*Lonicera japonica*	GST	Multicellular: peltate, capitate	Secretion of chlorogenic acid (antioxidant, medicinal)
*Ocimum basilicum*	GST	Multicellular: bulbous, capitate	Secretion of eugenol/linalool (antibacterial, spice)
*Phillyrea latifolia*	GST	Multicellular: peltate	Secretion of triterpenoids (stress responses)
*Cucumis sativus*	GST	Multicellular: conical	Secretion of cucurbitacins (anti-herbivory)
*Thymus vulgaris*	GST	Multicellular: peltate, capitate	Secretion of thymol (antimicrobial, medicinal)
